# The Indispensable Dog

**DOI:** 10.3389/fpsyg.2021.656529

**Published:** 2021-07-26

**Authors:** Clive D. L. Wynne

**Affiliations:** Canine Science Collaboratory, Department of Psychology, Arizona State University, Tempe, AZ, United States

**Keywords:** domestication, symbiosis, dominance, social hierarchy, dogs (*Canis lupus familiaris*), wolves (*Canis lupus lupus*)

## Abstract

Dogs’ remarkable success in living in a human-dominated world rests on a set of adaptations to cohabitation with humans. In this paper, I review the nature of these adaptations. They include changes in reproductive and foraging behavior from their ancestor species, wolves, which can be understood as adaptations to the change from hunting live prey to feeding on human food residues. Dogs also show several changes in social behavior which are more controversial and even somewhat paradoxical. Contrary to theories of canine domestication which view dogs as less aggressive and more cooperative than wolves, several studies show that dogs’ social interactions with conspecifics are more hierarchical and competitive than are wolves’. As scavengers rather than hunters, dogs do not need to cooperate with conspecifics the way that wolves do. But how then can we understand dogs’ willingness to cooperate with humans? I propose an integrated account of dogs’ social behavior that does not assume that dogs need to recognize the species-identity of the individuals with whom they interact. Because of the overlap in formal signals of dominance and submission between dog and human and people’s complete control over the resources dogs need, I propose that people occupy a status of “super-dominance” over dogs. This conception suggests several new lines of research which could shed light on the human-dog relationship to the benefit of both partners.

## Introduction

Dogs (*Canis lupus familiaris*) are by any measure exceptional beings. They are the most widespread large mammal (after humans) on this planet. The total world population of dogs is estimated to be around 800 million individuals (Hughes and Macdonald, 2013; Rowan, 2020), and dogs are present on every continent except Antarctica (there were dogs on Antarctica too until they were banned in 1994: British Antarctic Survey, n.d.). Dogs are the most phenotypically diverse mammal (Wayne, 1986) and were the first domesticated organism, arising from wolves (*Canis lupus lupus*) over 15,000 years ago – millennia before any other animal or plant was domesticated (Larson et al., 2012). Dogs live alongside people in a high state of intimacy. For example, over 50% of adult women respondents to a survey in the United States reported that they let their dogs sleep on their beds with them (Hoffman et al., 2018: it should be noted that the participants were self-selected and unlikely to be representative of the broader population; however, no better study is available). Some authors (e.g., Coppinger and Coppinger, 2002) have argued that this level of intimacy is a recent phenomenon, but, although the proportion of people living so intimately with dogs may have increased over the last two centuries (e.g., Ritvo, 1987), in a discussion of how to keep dogs written by an Ancient Greek nearly two millennia ago, the author advocates, “… it is best for (dogs) to sleep with men:- as they become thereby affectionately attached—pleased with the contact of the human body, and as fond of their bedfellow as of their feeder.” [Arrian, 1831, pp. 93–94 (original second century CE)]. The archeological record provides many forms of evidence that people and dogs have long lived in close connection with each other (Sykes et al., 2020).

Clearly, the remarkable success of dogs is due in some sense to their adaptations to human proximity. Dogs are obligatory human symbionts (Coppinger and Coppinger, 2002). That is to say, dogs are found living close by humans and dependent on food sources they obtain from humans. In the first world, this provisioning is mostly intentional: In the developing world, the provisioning is more often unintentional as when dogs scavenge on human refuse (Butler and du Toit, 2002; Coppinger and Coppinger, 2002). Few dogs survive entirely by hunting live prey, and there is no evidence of populations of dogs that are self-sustaining entirely by this method of foraging (Coppinger and Feinstein, 2015; Dingoes would be the one clear exception to this rule, if one considers dingoes to be dogs: Smith et al., 2019, but see also Jackson et al., 2019).

The importance of adapting to human proximity may be central to dog’s success in the human-dominated world, but the essence of that successful adaptation remains a topic of continuing debate and controversy in the literature. In this paper, I will restrict my discussion to aspects of dog behavioral adaptation to the human niche that are well established and consider what conclusions can be drawn from these facts.

## Reproductive Behavior

Modern dogs have notably more fluid reproductive behavior compared to their ancestors, wolves (*Canis lupus lupus*). Where wolves form pair bonds which can be lifelong, do not become reproductively active before the second year of life, have a rigid breeding season, and produce no more than one litter of pups per year (Rausch, 1967; Kleiman and Eisenberg, 1973; Macdonald and Moehlman, 1982; Haase, 2000; Mech, 2002), dogs are already reproductively active in their first year of life (Ghosh et al., 1984; Wandeler et al., 1993; Boitani and Ciucci, 1995; Lord et al., 2013). Female dogs may show preferences for certain mates and are not technically promiscuous, but they usually have multiple mating partners (Pal, 1999; Cafazzo et al., 2014). There is at least one report of male dogs guarding their mates through pregnancy and nursing (Pal, 2005), but in general, dog fathers do not contribute to the support of their mates or offspring (Lord et al., 2013 and references therein). Female dogs reproduce on average every 7 months throughout the year (Macdonald and Carr, 1995; Boitani et al., 2007), though seasonality in response to resource availability is possible, as in India, for example, where mating occurs in winter so that pups are born in the late monsoon season (Oppenheimer and Oppenheimer, 1975; Pal, 2001; Chawla and Reece, 2002; Pal, 2008). Unlike in wolves, males are continuously reproductively active (Gipson et al., 1975; Haase, 2000; Lord et al., 2013).

Both wolf parents collaborate to raise their pups; pups which may not leave their parents’ family group until the second year of life (Rausch, 1967; Mech, 1981; Peterson et al., 1984). By contrast, dog pups are nursed by their mother for 5 to 11 weeks (Martins, 1949, Scott and Fuller, 1974, Pal, 2001, 2008) and thereafter must survive on their own. There are sporadic reports of fathers regurgitating for their young (Malm, 1995; Pal, 2005; Paul et al., 2014) as well as playing and protecting them (Pal, 2005; Paul et al., 2014), but support from the father or young of earlier litters does not appear to be the norm (Martins, 1949; Mech and Boitani, 2003; Pal, 2008; Bonanni and Cafazzo, 2014). There are also reports of allonursing by females denning together (Daniels and Bekoff, 1989; Pal, 2005; Paul et al., 2014), but group denning does not appear to be widespread.

## Foraging Behavior

The quite distinct reproductive behaviors of wolves and dogs are clearly adaptations to their different foraging niches (Marshall-Pescini et al., 2017a). Wolves survive by hunting live prey which is larger than they are and is highly motivated not to become a wolf’s dinner. This can only be achieved by a close-knit group of individuals who have undergone a form of apprenticeship which can take from 1 to 3 years (Mech, 1981). Hunting for wolves is so complex that they tend to specialize on a subset of available prey species and interbreed preferentially among conspecifics who focus on the same prey species (Pilot et al., 2012). Wolf hunting success is dependent on group membership. For easier to kill prey, such as elk (*Cervus elaphus*), hunting reaches an optimum for groups of two to six wolves; for bison (*Bison bison*), which are far more challenging prey, capture success only levels off at groups sizes of 9 to 13 individuals (MacNulty et al., 2014).

The primary form of foraging for dogs is scavenging. The majority of the world’s dogs subsist on food remains discarded by humans (Boitani and Ciucci, 1995; Butler and du Toit, 2002; Bhadra, 2014; Coppinger and Feinstein, 2015), and even pet dogs fed directly by people are still technically scavenging in so far as the food given them is primarily either surplus to the human’s requirements or manufactured from “animal by-products” which are portions of meat animals that people prefer not to eat (What are animal by-products? n.d.). Dogs are not typically successful hunters and there are few populations of dogs which survive and maintain numbers entirely by hunting (Coppinger and Feinstein, 2015). The scavenging niche does not require the complex skillset that hunting live prey demands. It hardly needs noting that extracting and consuming the remnants of already deceased and butchered prey is a far simpler procedure and does not usually benefit from the coordinated action of a group of closely attached individuals. Indeed, the presence of conspecifics leads to competition in free-ranging dogs and they prefer to forage solitarily outside the mating season (Sen Majumder et al., 2014).

Dogs’ foraging and reproductive behavior can be understood as an interlocked suite of adaptations to a novel niche. Wolves need to reproduce seasonally because their prey shows seasonal availability. Dogs do not (typically) need to constrain themselves to only reproduce at particular times of year because the availability of their diet usually varies little by season. Wolves need to form pair bonds and keep their young with them to ensure their survival during the early months of life and then to apprentice them in the complex task of hunting large live prey. Their assistance is important in the success of the hunt (Mech, 1981; MacNulty et al., 2014). Dogs, on the other hand, can forgo pair bonding because their young require little training. Around 8 weeks of age, pups start following their mother to food sources and may also beg for food from people (Macdonald and Carr, 1995; Pal, 2008; Lord et al., 2013). No further parental support is offered. The more flexible reproductive strategy of dogs enables them to respond to sudden changes in resource availability such as when, for example, a new human group moves into their territory, or the foraging success of their host human population suddenly improves.

## Social Behavior

Both reproductive and foraging behavior include interaction with others and thus are forms of social behavior, but I now proceed to consider other aspects of social behavior in dogs. Like all social species, dog social behavior shows itself in interaction with conspecifics, but, unlike most species, dogs may also have important social interactions with members of other species. I consider these separately.

### Conspecific Behavior

The behavior of dogs toward others of their species does not consistently indicate strong within-species bonds. Dog pups show distress if forcibly separated from their mother (Fredericson, 1952; Pettijohn et al., 1977) but the only available investigation of the impact of the separation of adult kennel mates did not find any detectable impact on behavior or stress hormone levels (Tuber et al., 1996).

In free-roaming dogs, a diversity of social patterns has been found at different study sites around the world. Free-ranging dogs have been reported to be solitary or dyadic in studies from India: (Sen Majumder et al., 2014), Zimbabwe (Butler et al., 2004), the United States (Beck, 1973; Rubin and Beck, 1982; Berman and Dunbar, 1983, Daniels, 1983; Daniels and Bekoff, 1989), and Ethiopia (Ortolani et al., 2009). However, several studies have found dogs in groups ranging from 6 to 28 individuals in India: (Sen Majumder et al., 2014), Italy (Macdonald and Carr, 1995; Bonanni and Cafazzo, 2014), and the United States (Beck, 1973; Gipson, 1983). It appears that the size of groups may depend on the availability of food, the breeding status of females, and the season (Sen Majumder et al., 2014). Living in larger groups may offer protection (Bhattacharjee et al., 2020) and larger groups may also be more successful at hunting (Butler et al., 2004; Vanak and Gompper, 2009; Bhadra, 2014).

Although it should be noted that the studies cited here were carried out by different researchers in very diverse parts of the world and over a considerable time range – so that the range of findings may be due to different methodologies – nonetheless, there is suggestive evidence that dogs can adapt their social structure to suit changing circumstances.

#### Hierarchical Social Organization

Hierarchical social structure is a common, but not inevitable, concomitant of living in social groups (Immelmann and Beer, 1992; Dugatkin, 2020). The question of whether dogs live in hierarchical social groups, with the relativities of status for individuals which that implies, has become controversial in recent years because of the misuse of the term “dominance” by certain popular dog trainers, such as Millan and Peltier (2007), Fincke (2004–2016), and the Monks of New Skete (2002). These individuals use “dominance” as a cover for painful and regressive forms of animal training (Yin, 2007; American Veterinary Society of Animal Behavior, 2008; Bradshaw et al., 2009; McGreevy et al., 2012). This controversy has little to do with the use of “social dominance” in the strict ethological sense (Immelmann and Beer, 1992). Dominance in ethology is simply the tendency for certain individuals in a social group to have at least partially consistent preferential access to limited resources, such as shelter, food, and sexual partners (McFarland, 1987). Individuals with consistent access to constrained resources are known as “dominant”: Those that consistently have less access to resources are “subordinate.” Dominance hierarchies may form a consistent rank ordering, in which case Greek letters, alpha, beta, etc. are used to label individual positions with the hierarchy. The concept of dominance includes the enforcement of preferential access by aggression and agonistic interactions, but ethologists now also recognize that social hierarchies are commonly maintained by signals of superior and inferior status known as formal dominance and subordination signals (Peterson et al., 2002; Flack and de Waal, 2010). These formal signals are not in themselves aggressive or threatening but are understood by social interactors as indicating relative status, i.e., dominant or submissive.

Groups of free-living dogs have been found to live in social hierarchies in several studies including in Italy (Bonanni et al., 2010; Cafazzo et al., 2010; Bonanni et al., 2017; Silk et al., 2019), Spain (Font, 1987), and India (Pal et al., 1998; Sen Majumder et al., 2014). Social hierarchies have also been observed in owned dogs in the United States at a day care center (Trisko and Smuts, 2015) and a dog park (Bauer and Smuts, 2007). Furthermore, group-housed dogs studied in the Netherlands were found to experience social hierarchies (van der Borg et al., 2015).

The studies of free-living dogs in Italy found that dominant individuals had higher copulatory access (Cafazzo et al., 2014) and a higher likelihood of leading group movements than lower-ranking individuals (Bonanni et al., 2010). Silk et al. (2019) studying a group of 25 to 40 free-ranging dogs in a suburb of Rome, identified that older and male animals were typically dominant over younger female ones.

Two studies have identified formal dominance signals in groups of dogs. Bauer and Smuts (2007) studied owned dogs at a park and found that, even as the playing dogs reversed many roles – including chasing and tackling – certain behaviors remained stable in dyads. These including mounting, muzzle biting, and licking – suggesting they were stable formal dominance-status markers. van der Borg et al. (2015), studying a group of 16 dogs living in kennels with outdoor group play opportunities, noted two behavioral markers of formal dominance: high posture and muzzle bite. Several behaviors also functioned as formal markers of submission: body tail wag, lowered posture, mouth lick, and pass under the head. These authors analyzed the dominance structure of the dog group as a whole and, using a scale developed in primate research which categorizes social structures on a scale from (1) despotic through (4) egalitarian (Flack and de Waal, 2010), determined that the dogs scored around (2) tolerant. The dogs showed a moderately steep social hierarchy with large asymmetries in formal signal use and mild to moderate levels of aggression.

Bradshaw et al. (2009) argued that dogs do not form social hierarchies and presented data from a group of neutered males in which, they argued, no overall social structure could be observed. Notwithstanding this claim, the data presented clearly showed that at least some of the dogs formed a linear hierarchy of dominance status. Schilder et al. (2014), commenting on these findings, suggested that a group of human-resourced, sterilized, animals all of the same sex may have had no resources to compete over and thus might not be expected to show much overt social hierarchy.

Boitani and Ciucci (1995; see also Van Kerkhove, 2004; Boitani et al., 2007) also suggested that dog groups lack clear hierarchies because they observed multiple breeding individuals – which would not be found in a wolf pack. However, Cafazzo et al. (2010) noted that social hierarchies can still be present, including preferential reproductive access, even if the overall mating system of a group tends toward promiscuity.

Bradshaw et al. (2009) further raised the objection that “dominance” is often mistakenly spoken of, particularly by naïve dog trainers, as if it was a personality dimension – a property of an individual rather than of the interactions among individuals. Although it is true that dominance relations are defined by interaction, it is also the case that the nature of these interactions depends on certain relatively stable qualities of the interacting individuals. It is surely noteworthy that tests of dog personality or temperament currently in use have identified traits relevant to dominance and submission relationships. These include “submissiveness” (Jones and Gosling, 2005); “leader/dominant” (Ákos et al., 2014); and “boldness” (Svartberg et al., 2005).

The controversy over dominance in dogs is puzzling in so far as it has been known for many years that similarly raised groups of dogs show higher rates of conspecific aggression and competition than wolves (Frank and Frank, 1982; Feddersen-Petersen, 1991, 2007). Feddersen-Petersen (2004) even raised mixed groups of dogs (poodles) and wolves and found that, at 4 months of age, male poodles outranked the wolves in access to food and preferred locations.

More recent studies also show steeper social hierarchies in dogs than in wolves. Dale et al. (2016) gave similarly raised groups of dogs and wolves living in conspecific groups a carcass to feed on. Where subordinate wolves were able to feed to a similar level as their more dominant group-mates, dominant dogs monopolized the carcass at the expense of subordinate group members. Range et al. (2015) offered pairs of similarly raised dogs and wolves a food item that was large enough to be shared, but small enough to be monopolized by a dominant individual if it chose to do so. In the wolves, the dominant individuals tolerated their subordinate group-mates sharing food with them, whereas in dogs the dominant animals did not allow subordinate individuals to eat and subordinates did not even dare approach the food source.

#### Cooperation and Competition

In addition to their steep social hierarchies, dogs also show elevated levels of competition and have difficulty cooperating with conspecifics to solve tasks. Marshall-Pescini et al. (2017b) gave pairs of dogs and wolves from similarly raised groups a task in which the two animals had to pull on strings simultaneously for either of them to obtain a reward. Wolves were successful on the task but none of the dogs achieved any level of success. Ostojić and Clayton (2014) were able to demonstrate some success in dogs on this task by extensively pre-training the dogs. However, the dogs they tested were pets living together in human households where human intervention may have imposed levels of tolerance that the dogs left to themselves might not have developed (Marshall-Pescini et al., 2017b).

Bräuer et al. (2013, 2020) claimed to have demonstrated cooperation in pairs of pet dogs on a task where the dogs had to pass through one of two gateways in a barrier. However, this task is not a clear test of cooperation because each gateway was not wide enough or open long enough to permit two dogs to pass. Consequently, the dogs had to separate to pass through the gateways: One individual always had to wait for the other to pass through before its own gateway would open. Thus, the success of dogs on this task is in fact evidence of their reluctance to cooperate – in the strong sense of come together to work on a task together – rather than the opposite.

Although it may run against expectations based on interaction with household pets, there is abundant evidence in the scientific literature that dog groups can be very hierarchical, and dogs may be highly competitive and reluctant to share resources. Marshall-Pescini et al. (2017a) pointed out that, relative to their ancestors, wolves, dogs have less need to cooperate in their foraging and also cooperate less in raising young. Wolves have an essential need to cooperate with group members in order to kill the prey on which they feed. Furthermore, the outcome of a successful hunt is usually more than an individual wolf can consume. Consequently, wolves have many motivations to cooperate in foraging and sharing the results of their kill. These factors that motivate cooperation in wolves have limited applicability for dogs who have less need either to cooperate in obtaining food or to share the results of their foraging.

Overall, there is plentiful evidence that the social structure of dogs is both more flexible than that of wolves, with groups varying in size from solitary individuals to more than two dozen, but also shows signs of more extreme social hierarchy. This flexibility of social group size presumably reflects the diversity of food sources and dangers that dogs face in different parts of the world (as well, possibly, as different study methods). The steeper social hierarchy found in dogs than wolves is more surprising and even counter-intuitive but may also be related to dogs’ foraging strategy where cooperation is seldom needed, often counter-productive, and may have been selected against. To date, there do not appear to be any studies on the genetic relatedness of individuals within dog groups that might address the possibility of kin selection for altruistic and cooperative behavior.

### Heterospecific Behavior

Dogs not only have social interactions with their own species but also can form social groups with members of other species including, most particularly, human beings.

#### Flight Distance

One simple behavioral measure of dogs’ tolerance for human proximity is assayed as flight distance – the linear distance at which an individual flees from a gradually approaching human. Dogs reduced flight distance compared to wolves is surely a major component of their adaptation to living in proximity to humans and scavenging on human food remnants. For animals foraging on human trash dumps, flight distance to the approach of humans will be a key determinant of their extractive effectiveness.

Flight distance is defined as the distance from an intruder at which an individual flees (Immelmann and Beer, 1992). Wolves scavenging on human refuse in Scandinavia have been observed to have a flight distance to the human approach of around 200 m (Karlsson et al., 2007). Estimates of flight distance in dogs are quite varied, but all are considerably shorter than this estimate for wolves. Bonanni and Cafazzo (2014) reported flight distances of 20–50 m in free-ranging dogs in Rome, Italy. Ortolani et al. (2009) reported flight distances of around 5 m in free-ranging dogs around villages in rural Ethiopia. Although no formal data appear to be available, everyday experience indicates that the flight distances of pet dogs living in human homes are less than 1 m – if the concept of flight distance can be applied to these animals at all.

#### Attachment to Humans

As pet dogs are commonly spoken of as family members or friends to humans (Serpell, 2004), several investigators have adapted measures that are commonly used to study intimate relationships in human psychology to the study of dog-human relationships. Several studies have used a modification of a procedure commonly used to measure the strength of attachment between a child and his or her primary caregiver (usually the mother) – the strange situation procedure (SSP) developed by Ainsworth et al. (1970). In this test, a child is brought into an unfamiliar room with his mother. The child is briefly left in the room with a stranger; the mother returns, comforts the child and then leaves with the stranger so the child is briefly completely alone. Finally, the stranger returns, followed by the mother. Attachment is categorized on the basis of how the child reacts to being left alone and with the stranger and how he responds to being reunited with his mother (Ainsworth et al., 1978). Securely attached children are those who are happy to explore in their mother’s presence and are distressed by her disappearance but show a willingness to be comforted quickly on her return.

Several studies, starting with Topál et al. (1998), have shown that many dogs tested in the SSP with their primary caregivers show secure attachment toward the humans they live with (e.g., Topál et al., 1998; Rehn et al., 2013; Thielke and Udell, 2019, 2020; Wanser and Udell, 2019; Wanser et al., 2020). Two additional observations in the SSP raise questions about how to understand this finding, however. First, Gácsi et al. (2001) found that dogs living in an animal shelter tested in the SSP with a person they had only interacted with three times for 10 minutes per session showed clear signs of attachment toward that person. Second, the only study that tested dogs in the SSP with another dog from the same household as “caregiver” (Mariti et al., 2014) found few signs of distress when the target dog was separated from its companion, and these dogs were, in fact, less stressed when left alone with an unfamiliar person than when they were in the company of the other dog.

A handful of studies have investigated hand-reared wolves’ reactions to separation and reunion with familiar humans in the SSP. Topál et al. (2005) tested a group of hand-reared wolves at 16 weeks of age alongside a group of pet dogs of the same age. These authors found no signs of attachment to human caregivers in the wolves. In contrast, when Hall et al. (2015) tested hand-reared wolf pups at 3, 5, and 7 weeks of age, they found clearly differentiated responses to caregivers compared to strangers and strong responses to the reunion after separation, leading them to conclude that their wolf pups were securely attached to the caregivers. This pattern of results might suggest that hand-reared wolf pups show attachment to caregivers that fades as they grow older; however, Lenkei et al. (2020) tested adult wolves in the SSP and found secure attachment to human caregivers. Hall et al. (2015) suggested that Topál et al. (2005)’s failure to find secure attachment might have been due to the fact that the animals they tested were permanently removed from human homes between 2 and 4 months of age.

Taken together, the findings from hand-reared wolf pups and dogs tested in the SSP suggest that dogs may form secure attachments to human caregivers, but more rapidly than would be expected in our own species. Wolves may also under certain conditions show secure attachment, but in their case, the conditions for this finding may be more limited. However, the restricted range of studies on hand-reared wolves means these conclusions must be approached with caution.

Other, somewhat simpler, tests have also demonstrated pet dogs’ interest in their owners. Horn et al. (2013) presented pet dogs with a manipulative problem and compared how long they attempted to solve the task either with their owner in the room with them or on their own. The presence of the owner prompted the dogs to persist longer with the task than when left alone. Gácsi et al. (2013) found that dogs were less stressed when a stranger approached if they were with their owner than when alone. No equivalent tests appear to have been carried out on hand-reared wolves.

Jakovcevic, Mustaca, and Bentosela (2012) studied the bond between dog and human simply by measuring the latency to approach and proportion of a two-minute interval a dog would spend within 1-m of a seated person. Bentosela et al. (2016) extended this paradigm to hand-reared wolves and found that dogs had a considerably shorter latency to approach both familiar and unfamiliar seated humans than wolves and also spent more time within 1-m of the person.

Findings that pet dogs are disturbed by the sound of a human crying (Custance and Mayer, 2012; Yong and Ruffman, 2014) and will attempt to rescue their apparently trapped owner (Bourg et al., 2020) may also be viewed as evidence that pet dogs can become emotionally attached to people.

#### Cooperation With Humans

A variety of studies demonstrate that dogs readily attend and respond to human behavior. Pet dogs have been shown to beg from people who can see them in preference to people whose vision has been obscured in certain ways (Cooper et al., 2003; Gácsi et al., 2004; Udell et al., 2011). Udell et al. (2011) found that dogs only attended to forms of visual occlusion with which they had prior experience and hand-reared wolves were also sensitive to the implications of certain forms of obscuring of human vision. To date, studies of this type have not been attempted on dogs that were not living as pets in human households.

Wolves have been compared to dogs in tests of cooperation involving pulling on strings to obtain food. In studies, where food can only be obtained when two partners pull simultaneously on opposite ends of a string, hand-reared wolves have shown similar levels of cooperation with human partners as dogs (Range et al., 2019).

Several studies have demonstrated that pet dogs will follow human pointing gestures to find hidden food (e.g., Hare et al., 2002; Hare and Tomasello, 2005; Bräuer et al., 2006; Udell et al., 2008; Kaminski and Nitzschner, 2013). This ability has also been demonstrated in hand-reared wolves (Udell et al., 2008; Gácsi et al., 2009), and a recent review identified a wide range of both domesticated and non-domesticated species from diverse taxa which follow human pointing gestures given prior experience around people (Krause et al., 2018).

Dogs not living as pets in homes do not show the same level of success in following human pointing gestures. Reduced performance in the following points has been observed in street dogs in India (though see also Bhattacharjee et al., 2017, 2020, for evidence of successful point following in about half the street dogs approached), as well as kennel-living dogs (Udell et al., 2010; Lazarowski and Dorman, 2015).

The fact that dogs’ success in attending to and following human actions depends on the individual dog’s experiences around people, combined with the plentiful evidence that individuals from a wide range of species can also follow human gestures if they have had suitable ontogenetic experiences, indicates that dogs’ readiness to cooperate with people is a consequence, rather than a cause, of their success in living alongside humans.

#### Summary on Heterospecific Behavior

Dogs’ interactions with humans can be classified into two groups: the more emotional, attachment-like, patterns of behavior and the more cognitive or conditioned responses to specific human actions, such as pointing gestures. Emotional responses, including fear reactions as measured in flight distance, show differences between dogs and wolves with dogs much less fearful and more likely to form attachments to people than are wolves. On the other hand, reactions to discrete human actions do not appear to show the same kinds of differences between dogs and wolves.

## Conclusion: Dogs’ Adaptations To Humans

Dogs’ enormous success living in a human-dominated world rests on a set of adaptations to living in close proximity with our species. These include alterations in reproductive and foraging behavior from their ancestor species, wolves, which are readily understood as adaptations to the change from hunting live prey to scavenging on food residues that people offer – whether intentionally or not. The changes in dog social behavior are less obvious and indeed somewhat paradoxical. Contrary to theories of canine domestication which propose that dogs are less aggressive and more cooperative than wolves (e.g., Hare and colleagues’ “Survival of the friendliest,” Hare et al., 2002; Hare, 2017; Miklósi and Topál’s “Inter-specific social competence” hypothesis Miklósi and Topál, 2013), in fact, several studies clearly show that dogs, in their interactions with members of their own (sub) species are in fact more competitive and aggressive than are wolves. A strict social hierarchy may be even more important to dogs since their food is often in small portions that cannot be shared, unlike the larger carcasses on which wolves often feed.

In itself, dogs’ more competitive and hierarchical interactions with their own species are not inconsistent with their foraging niche. Dogs do not share wolves’ need to cooperate to obtain or consume food (Marshall-Pescini et al., 2017a). Placed alongside dogs’ willingness to attend and cooperate with humans, however, it does present a paradox of sorts: How to conceive of dogs’ different patterns of social behavior toward their own species on the one hand and humans on the other? It is implausible to propose that dogs have different programs of social behavior that they bring into play depending on the species identity of the social partners they are interacting with because no mechanism of species identification has ever been proposed. No mammal is born recognizing its own species – rather it develops an awareness of what kinds of individuals to have social relationships with during the critical period for social imprinting early in life (Hess, 1973). Furthermore, dogs do not just have social relationships with conspecifics and humans: They may also form social bonds with members of other species they interact with during the critical social imprinting period (Coppinger and Coppinger, 2002). Thus, livestock guarding dogs raised alongside sheep or goats will socially imprint on those species and socially interact with them through life. How does a dog know whether it should interact with sheep competitively – as it would with another dog, or cooperatively – as it would with a human? Clearly some more over-arching explanation is needed that does not assume that dogs identify diverse species and bring different social behavior patterns into play depending on that identification.

Range et al. (2019) suggested that dogs’ behavior toward humans could be viewed as “deferential” and that this is then consistent with what they view as a “conflict-avoidant” pattern of social interaction with conspecifics. I have taken this valuable suggestion further and proposed that dogs’ extreme sensitivity to hierarchy in social relationships may be the solution to the apparent paradox of their different behavior toward humans and conspecifics (Wynne, 2021). Several of the formal indicators of dominance and subordinate status in dogs overlap with behaviors used in the same way by humans (Schilder et al., 2014). Thus, van der Borg et al. (2015) identified high posture and muzzle bite as formal dominance indicators in dogs, along with low posture, passing under the head and mouth lick as submission indicators. In humans, raised posture has been noted as a dominance indicator (Mignault and Chaudhuri, 2003), along with sitting straight up (Schwartz et al., 1982) and raised head (Carney et al., 2005). Lowered head and other forms of lowered posture, such as kneeling, along with kissing, are formal markers of submission in humans (Kalma, 1991; Mignault and Chaudhuri, 2003).

Consequently, when people stroke dogs’ heads, accept licks near the mouth and make themselves taller than dogs they are unconsciously expressing formal dominance over their dogs. Combined with human’s total control over the resources that matter to dogs, such as food, freedom of movement, access to shelter, and even mating opportunities, this establishes dogs in a state of utter subordination to humans. Tinbergen (1969) proposed the concept of a supernormal stimulus, a stimulus that does not occur in nature but which exaggerates the features of naturally occurring stimulus and thereby evokes an exceptionally strong response. I proposed, by analogy to the supernormal stimulus, to call the relationship of human to dog “super-dominance” because no conspecific could possibly control a dog’s access to resources to the extent a human does (Wynne, 2021).

The relationship of dominance offers a mechanism for dogs to respond differently to members of different species without any need to propose that dogs identify the species to which individuals belong. A dog’s social behavior toward individuals from other species would depend on the extent to which the individual expresses behaviors the dog recognizes as dominant to itself along with the individual’s control over resources of importance to the dog (as well as human intervention to control the dog’s behavior towards a third species).

This concept of super-dominance bears no relationship to the confused notions of “dominance” espoused by certain currently popular dog trainers, such as Millan and Peltier (2007), Fincke (2004–2016), and the Monks of New Skete (2002). What these trainers mean by “dominance” is closer to concepts of positive punishment and negative reinforcement. Indeed, the “positive” trainer who controls an animal’s behavior with contingent treats, strokes her dog’s head and allows it to “kiss” her, is expressing dominance over her dog to a greater degree than the misguided person who imagines dominance is conveyed by always walking through a doorway first (Millan and Peltier, 2007).

This conception suggests several lines of research which may contribute to better lives for people and their dogs. For example, it is very striking that although there are now a few ethological studies of free-ranging dogs, there are almost no studies of how people and dogs live alongside each other in homes. If, as I propose, dogs’ lives with people are structured around dominance relationships, dogs should react differently toward people who express different levels of dominance. Dogs would be predicted, for example, to respond differently toward people of different levels of stature, toward people with differing levels of control over resources that matter for dogs, and so forth. At present, even the most basic observational facts about how dogs and people live together are strangely absent from the literature. For example, we do not know how much time pet dogs spend in proximity to the humans in their household, what form the interaction takes nor how this depends on age, sex, breed of dog, or cultural background of the person. Consequently, the many observations that people feel affection for their dogs and the apparent reciprocation of that emotion by dogs have not been set into a context of objective measurement of behavioral interaction. Whatever the value of the super-dominance hypothesis, studies of this kind could shed light on and offer to improve dogs’ lives in human society.

## Data Availability Statement

The original contributions presented in the study are included in the article and further inquiries can be directed to the author.

## Author Contributions

The author confirms he is the sole contributor to this work and has approved it for publication.

## Conflict of Interest

The author declares that the research was conducted in the absence of any commercial or financial relationships that could be construed as a potential conflict of interest.

## Publisher’s Note

All claims expressed in this article are solely those of the authors and do not necessarily represent those of their affiliated organizations, or those of the publisher, the editors and the reviewers. Any product that may be evaluated in this article, or claim that may be made by its manufacturer, is not guaranteed or endorsed by the publisher.
